# Playing Tetris Lets You Rate Odors as Less Intense

**DOI:** 10.3389/fpsyg.2021.657188

**Published:** 2021-07-14

**Authors:** Stephan Schadll, Rea Rodriguez-Raecke, Lennart Heim, Jessica Freiherr

**Affiliations:** ^1^Diagnostic and Interventional Neuroradiology, RWTH Aachen University, Aachen, Germany; ^2^Department of Psychiatry and Psychotherapy, Friedrich-Alexander-Universität Erlangen-Nürnberg, Erlangen, Germany; ^3^Sensory Analytics, Fraunhofer Institute for Process Engineering and Packaging IVV, Freising, Germany

**Keywords:** olfaction, smell, attention, distraction, cognitive load

## Abstract

Overweight and obesity are considered a huge problem in modern societies. Previous studies have shown that people who are regularly distracted by playing videogames or watching TV while eating are more likely to be overweight and that the number of people that are gaming worldwide is rising. Further, it has been established that both, watching TV or playing video games lead to an increased snack intake and a lower rating of perceived taste intensity. Since flavor perception is accomplished not only by the sense of taste but also the sense of smell, we investigated the influence of cognitive load created by playing a video game on odor intensity perception. The participants played a low or high difficulty version of Tetris while presented with odors of food and non-food items. A higher skin conductance response (SCR) along with a decrease in task performance verified that the higher difficulty level leads to a higher cognitive load. Our behavioral data indicates a significant decrease in intensity estimates of food odors and non-food odors during the high compared to low cognitive load condition. We conclude that odor intensity estimation is influenced by real-life cognitive tasks which might in turn lead to overeating while distracted.

## Introduction

Overweight and obesity are a growing problem for adults, as well as for children. The number of obese and overweight people worldwide in 1980 was 857 million (Wise, 2014). That number went up to over 2.1 billion people in 2014 indicating an increase of 28% among adults and 47% increase among children (Wise, 2014). The prevalence of obesity and overweight in developed countries in childhood especially increased between 1980 and 2013, from 17 to 24% for boys and from 16 to 23% for girls (Wise, 2014).

The reason for this development is our obesogenic environment with the main factors being an energy intake that excesses the energy needs, calorie-dense, nutrient-poor food choices, and low physical activity (Hruby and Hu, 2015). A study by Melchior et al. (2014) indicated that young adults aged 22–35 years who play video games on a regular basis are more likely to be overweight. Furthermore, a study by Braude and Stevenson (2014) led to the conclusion that people who regularly watch TV while eating have a higher snack intake in comparison to people who do not regularly eat while watching TV. Playing a video game leads to an acute stress response that is associated with increased food intake (Siervo et al., 2018), and the number of people playing video games, also called gamers, is rising. In 2018, there were about 2.3 billion gamers worldwide who spent about $137.9 billion on games with mobile gaming being the largest segment (51%), followed by console gaming (25%) and PC gaming (24%) (Wijman, 2018). In 2019, the number of gamers has increased to 2.5 billion who spent about $152.1 billion on games (Wijman, 2019). While mobile gaming remained the largest segment, taking up 45% of the global games market, console gaming grew to 32% of the global games market and PC gaming accounted for 23% of the global games market (Wijman, 2019). Our study aims to explore if playing video games can alter the way we perceive odors which could be a reason for the increased food intake.

An experiment by van der Wal and van Dillen (2013) used a memory task to simulate cognitive load. In this memory task, the participants had to remember either digits or consonants. The authors showed that this workload task led to a decreased taste intensity perception. This decreased intensity perception further guided subjects to choose taste solutions with higher concentrations to compensate and to consume more of those taste solutions. Other studies by Bolhuis et al. (2011); Ramaekers et al. (2014), and Jayasinghe et al. (2017) showed that the taste intensity of different food items affected the food intake, with higher intensity resulting in a lower food intake. Since food consumption is a multisensory process and olfaction is an important component of taste perception (Murphy et al., 1977), another study examined the effects of the same memory task used by van der Wal and van Dillen (2013) on the intensity perception of odors (Hoffmann-Hensel et al., 2017). This study from our lab showed that a high cognitive load resulted in a decrease in odor intensity perception of low-caloric but not high-caloric food items.

Based on these experiments, we aimed for a more ecologically valid distraction task to explore if an everyday high cognitive load task leads to a decrease in intensity perception of different odors. We selected the video game Tetris because of its simplicity and to minimize an emotional response to the game. It is cognitively demanding and requires strategic planning, prediction of future game situations, mental rotation, manual dexterity, and decision-making in real time (Lindstedt and Gray, 2015). The Tetris game has already been included in other research studies (Lau-Zhu et al., 2017).

A couple of studies have shown that cognitive load leads to physiological changes that can be detected by analyzing the skin conductance response (SCR) (Jacobs et al., 1994; Shimomura et al., 2008). Therefore, we measured SCR to verify the efficiency of our cognitive load task alongside with analyzing the behavioral changes on the cognitive load conditions. We expected to see increased SCR values during the high cognitive load compared to the low cognitive load condition.

To differentiate if a cognitive load task induces a change in intensity perception of all types of odors or if there is a difference between food and non-food odors, we investigated both. Based on the findings of Hoffmann-Hensel et al. (2017), we expected our cognitive load task to be sufficient to produce a decrease in intensity perception for food as well as for non-food odors.

## Materials and Methods

The RWTH University Aachen Medical Faculty local ethics board approved the current study (approval number: EK 224_11). Our study protocol complies with the Declaration of Helsinki for Medical Research involving Human Subjects. A written informed consent and a data security statement was signed by each participant.

### Participants

#### Inclusion Criteria

Prior to the screening, the participants had to perform the Beck Depression Inventory (BDI) (Beck et al., 1961) and the Brief Symptom Inventory (BSI) (Derogatis and Melisaratos, 1983) to control for psychiatric disorders. Thirty out of 31 participants fulfilled the common inclusion criteria for BDI and BSI (BDI < 13, inclusion for the BSI was based on the SCL-90-R norm values controlled for age and sex, with a Global severity index, which reflects the overall psychological stress, below 64). The remaining 30 physically healthy, self-reported non-smokers [17 females, mean age = 24.0 years; standard deviation (SD) = 3.4 years; and range = 18–32 years] with a BMI between 18.7 kg/m^2^ and 29.6 kg/m^2^ (mean = 23.0 kg/m^2^; SD = 2.5 kg/m^2^; and cut-off value = 30) were tested with no exclusions. The MONEX-40 (Freiherr et al., 2012), which includes 40 common odors in a four-alternative, forced-choice identification task, was used to confirm normosmia of the subjects (mean = 33.1; SD = 2.0; range = 28–37, and cut-off value = 27). Furthermore, the participants had to take the Montreal Cognitive Assessment (MOCA) (Nasreddine et al., 2005) test to control for cognitive abnormalities. All 30 participants fulfilled the common inclusion criteria of the MOCA (≥26/30).

#### Instructions to the Participants

Participants were instructed not to use any nasal sprays or any strong-smelling cosmetics like perfume or deodorant on the testing day. Moreover, they were instructed not to eat or drink anything besides water 1 h prior to testing (Stafford and Welbeck, 2011; Hanci and Altun, 2016).

Those screening and preparation steps as well as the exclusion criteria are similarly used in studies that incorporate smell in human computer interaction (Obrist et al., 2014).

### Olfactory Stimulation

#### Odor Selection

For the two odor categories, two respective odors were selected, as well as a no odor stimulus (water). All five stimuli were presented in a random order. Food odors included banana (banana flip from Burghart Messtechnik GmbH, Wedel, Germany; 20% in propylene glycol (PG) from Sigma, product number 82280) and orange (orange oil from Pajoma, product number 91356, 25% in diethyl phthalate (DEP) from Sigma, product number 524972) and non-food odors included leather (shoe leather from Burghart Messtechnik GmbH; 25% in DEP) and wood [(1R)-(+)-α-pinene from Sigma Aldrich, product number W290238, 1.5% in PG]. In a pilot study, we presented these odors to 13 healthy subjects and asked them to rate the intensity of the odors on a scale of 1–10 (1 = low intensity, 10 = high intensity). The odor presentation in the pre study was accomplished by presenting a small glass jar containing the respective odor solution to each participant. The data revealed that there is no difference in intensity perception between these odors, when the subjects are not experiencing a cognitive load task [*F*_(__3,__51)_ = 0.618, *p* = 0.607]. In this pilot study, we also collected data to ensure the food odors are perceived as edible and the non-food odors are perceived as inedible. Therefore, the subjects rated the edibility of the odor on a scale from 1 to 10 (1 = not edible, 10 = edible). These scales from 1 to 10 for edibility have been used in experiments before for example by Hudry et al. (2002). We tested the ratings of our subjects using one sample *t*-tests with the test variable being the odor ratings against a test value of 5. The test revealed that the banana odor (mean = 9.23, SD = 1.09) was perceived as an edible odor [*t*_(13)_ = 13.97, *p* < 0.001] just like the orange odor [mean = 8.23, SD = 1.74, *t*_(13)_ = 6.7, and *p* < 0.001]. Moreover, the leather odor [mean = 1.23, SD = 0.6, *t*_(13)_ = −22.7, and *p* < 0.001] as well as the wood odor [mean = 1.92, SD = 1.19, *t*_(13)_ = −9.34, and *p* < 0.001] were perceived as inedible.

#### Odor Application

The odors were administered using a computer-controlled olfactometer (Lundstrom et al., 2010) with a constant air flow of 3.0 l per minute. The odors were presented in a block design with 2 s on and 2 s off blocks alternating for 28 s. In total, four odors and a no-odor stimulus (water) were applied to both nostrils. Each odor was presented eight times, four times in a low cognitive load condition and four times in a high cognitive load condition for a total of 112 s per odor. Overall, the participants were presented an odor or the control condition for 560 s.

### Experimental Design

#### Experimental Setting

The experiment was designed and executed in PsychoPy 2 (University of Nottingham) (Peirce, 2008). The tasks were presented on two computer screens. The right screen displayed instructions and the rating scales while the window with the Tetris game appeared in the top left corner of the left screen. The participants used a keyboard for their responses. They only used the right hand to operate the keyboard.

#### Cognitive Load Task

**FIGURE 1:**
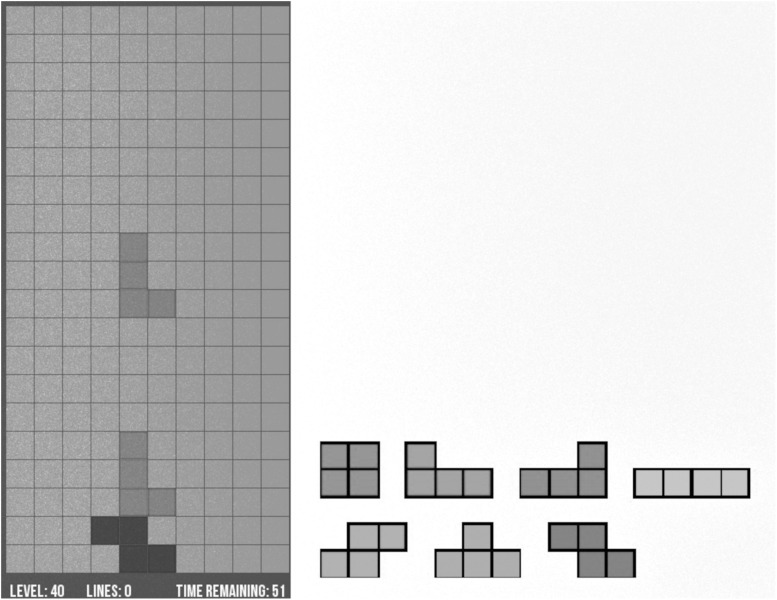
Depiction of the Tetris game and the blocks that were used.

Before starting the cognitive load task, the participants had to put on noise canceling headphones. The cognitive load task consisted of playing Tetris, which was programmed using a Python script by Mr. Loeber, who gave permission to use his work for our experiment^1^. The playing field had 10 by 20 squares and we used seven different blocks, each block having four squares arranged differently (Figure 1). Two different levels were used to represent low and high cognitive load with speed being the difference between the levels. During the low cognitive load condition, the blocks dropped down one square every 0.47 s as compared to every 0.1 s during the high cognitive condition. The participants used the keyboard to move the Tetris blocks. By pressing the down-arrow key, the Tetris block instantly dropped down one square and by pressing space bar, the Tetris block instantly dropped to the lowest position possible. The participants played for 56 s and had to solve as many rows as they could. If the participant reached the top of the playing field within those 56 s, the playing field would clear so the participant could keep on playing.

#### Odor Stimulation

In the first 28 s, the participant played the game, and no odor was presented. This way, the participant got used to the task and was already situated in the low or high cognitive load condition when we started applying the odor. After the first 28 s, we started to administer the odor in a block design for 28 s (2 s odor on, 2 s odor off) until the game stopped.

#### Feedback of the Participants

**FIGURE 2:**
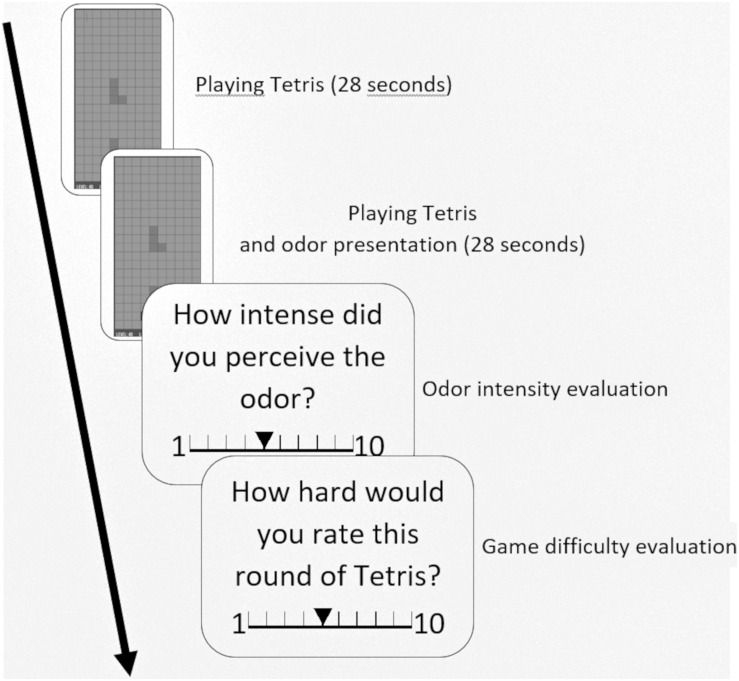
Flow-chart of the experiment. The subjects first played Tetris for 28 s, then the odor was presented while they kept playing Tetris for another 28 s. Then the subjects were asked to rate the intensity of the odor and the game difficulty on a scale from 1 to 10.

When the game was finished after 56 s, the window closed itself and the participants were asked to rate the intensity of the odor that was presented in the last round on a scale from 1 to 10, 1 indicating a low intensity and 10 a high intensity. After the intensity rating, the participants were also asked to rate the difficulty of the last round on a scale from 1 to 10, 1 indicating a low difficulty and 10 a high difficulty (Figure 2). The participants chose the number with the left and right arrow keys on the keyboard and confirmed their choice by pressing the space bar.

#### Experiment Duration

In total, the participants played 40 rounds of Tetris, 20 rounds with low difficulty (four rounds for each of the four odors and four rounds for the no-odor condition) and 20 with high difficulty (four rounds for each of the four odors and four rounds for the no-odor condition). Each round was 56 s long for a total of 37 min and 20 s of playing Tetris. The order in which the odors were applied as well as the order of the difficulty levels were randomized.

#### Skin Conductance Response Measurement

The skin conductance (SC) data was acquired in microsiemens (μS) using the Powerlab 8/35 system (ADInstruments Ltd., Oxford, United Kingdom). We used a FE116 GSR Amp (ADInstruments Ltd.), which according to ADInstruments specification, is a “75 Hz oscillator which supplies a near square wave, low-impedance, low-voltage (22 mVrms) signal to an electrode on one finger of the subject” (ADInstruments, 2020). The electrodes used for acquiring the SCR data (GSR Finger Electrodes (MR Safe), MLT117F, ADInstruments) were placed on the palmar surface on the middle phalanx of index and middle finger of the left hand with an electrode gel [Electrode gel, isotonic, 114 G (0.05M NaCl), Gel101, Biopac Systems, Inc.]. The program we used to record the SCR data acquisition was LabChart 8 (ADInstruments). Prior to starting the first round of Tetris, we collected 5 min of SC data without the participant stimulation as a baseline for each subject which is needed to determine the tonic activity (Benedek and Kaernbach, 2010b).

### Statistical Analysis of Behavioral Data

The behavioral data was analyzed using SPSS 25 (IBM, Ehningen, Germany). The statistical approach for the behavioral data was chosen based on the hypotheses we developed before we started testing our subjects. All data was tested for homogeneity of variance using the Levene test. We transformed data on the number of rows solved, using a common logarithm to achieve a normal distribution. The impact of cognitive load and edibility on the subjectively reported odor intensity was tested using a maximum likelihood linear mixed model (LMM) with the fixed factors cognitive load (low/high) and edibility (food/non-food) and a random factor for subject. Our control condition was tested using a dependent *t*-test with the intensity ratings for the no odor trials for each cognitive load condition (low/high) as outcome variables. We used a dependent *t*-test with the average intensity rating of the first 20 trials and the last 20 trials for each participant as outcome variables to test, if the participants adapted to the stimulation and showed a decrease in odor intensity perception in later trials. Furthermore, we used a Pearson correlation to evaluate a possible correlation between BMI and intensity ratings. A maximum likelihood LMM with the variable difficulty ratings, the fixed factor cognitive load (low, high) and a random factor for subject was used to test for the influence of cognitive load on the difficulty ratings and another maximum likelihood LMM with the variable number of rows solved, the fixed factor cognitive load (low, high) and a random factor for subject was used to test for the influence of cognitive load on the number of rows solved. The threshold for all tests was set to *p* < 0.05.

### Statistical Analysis of SCR Data

We chose the statistical approach for the SCR data based on the hypotheses we developed before we started testing our subjects. The SCR we collected in LabChart was merged with the PsychoPy files for each subject to synchronize the events with the physiological data.

We downsampled the SCR from 1,000 to 100 hertz (Hz) using Matlab (MathWorks^®^, Aachen, Germany) for better computing performance. Afterward, we used Ledalab^2^ for the analysis of our SCR data. We used the approach Benedek and Kaernbach (2010a, b) described to analyze our SCR data. They used a continuous decomposition analysis (CDA) which separates the SCR data into continuous signals of phasic and tonic activity. The tonic activity reflects the slow varying SC level when no stimulus is applied. It can be estimated by measuring SC data of the participants without them being stimulated as a baseline. The phasic activity is the vast-varying activity with a zero baseline and event-related changes that portray responses specific to a stimulus and non-specific response. We then conducted an event-related analysis of the phasic activity by inserting the time stamps of the Tetris game start – each start of a Tetris game was an individual event.

The statistical analysis was performed using SPSS 25 (IBM, Ehningen, Germany). As a dependent variable we used the mean SC value of the phasic driver within the response window (global mean) in a 10 s time interval starting 0.8 s after the start of the odor application for each round. We tested for an impact of the cognitive load setting (low, high) on the global mean value using a LMM with the fixed factor cognitive load (low, high) and a random factor for subject. The threshold for this test was set to *p* < 0.05.

## Results

### Odor Intensity

**FIGURE 3:**
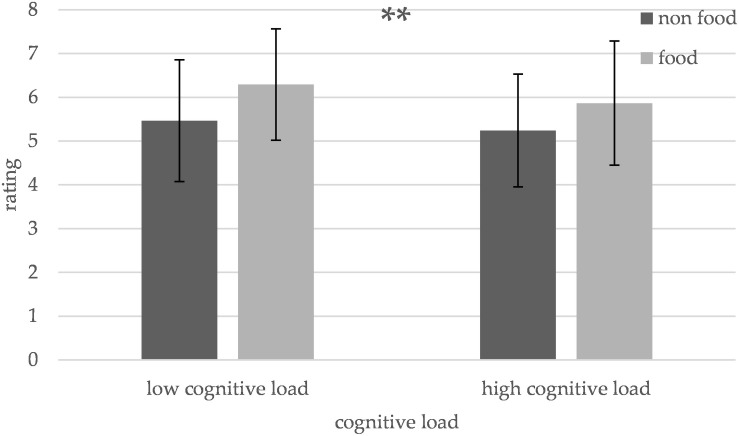
Influence of cognitive load on the intensity ratings for different odors. The intensity ratings of food and non-food odors decrease under high compared to low cognitive load [**p < 0.01, error bars portray one standard deviation (SD)].

The tests showed a significant main effect of cognitive load [*F*_(__1_,_90__)_ = 7.97, *p* = 0.006] and edibility [*F*_(__1_,_90__)_ = 39.64, *p* < 0.001] on odor intensity ratings. The interaction between edibility and cognitive load did not reach significance [*F*_(__1_,_90__)_ = 0.75, *p* = 0.387]. The intensity ratings for food odors decreased from low (mean = 6.29, SD = 1.27) to high cognitive load (mean = 5.87, SD = 1.42, and *p* = 0.011), just like the intensity ratings for non-food odors decreased when low (mean = 5.47, SD = 1.39) and high (mean = 5.24, SD = 1.29, and *p* = 0.170) cognitive load conditions are compared, however, this comparison did not reach statistical significance (Figure 3). Food odors were rated significantly more intense (mean = 6.08, SD = 1.35) than non-food odors (mean = 5.35, SD = 1.33, and *p* < 0.001). Further, there was no significant effect of cognitive load on the no odor condition [*t*_(1,__29__)_ = 0.77, *p* = 0.447] (Figure 3). Moreover, there was a significant negative correlation between the odor intensity ratings and the BMI of the subjects (*r* = −0.495, *p* = 0.005).

There was no significant difference in odor intensity ratings [*t*_(1__,29)_ = 1.75, *p* = 0.09] between the earlier (mean = 5.54, SD = 1.22) and the later (mean = 5.2, SD = 1.38) trials indicating that no adaptation to the odors occurred during the experiment.

### Cognitive Load

**FIGURE 4:**
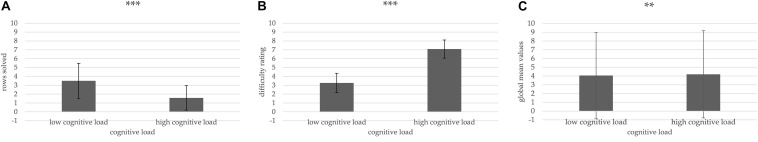
Influence of cognitive load on number of rows solved, difficulty ratings and Global Mean values. The number of rows solved decreased during the high compared to low cognitive load condition **(A)**. Difficulty ratings increased under the high compared to low cognitive load condition **(B)**. The Global Mean values of skin conductance response (SCR) increase with high compared to low cognitive load **(C)** (**p < 0.01, ***p < 0.001, error bars portray one standard deviation).

There was a significant effect of cognitive load on the number of rows solved [*F*_(1,__30__)_ = 86.503, *p* < 0.001] and the subjectively reported difficulty evaluation [*F*_(1, 30)_ = 306.625, *p* < 0.001]. The number of rows solved was significantly lower during the high (mean = 1.57, SD = 1.38) compared to the low cognitive load condition (mean = 3.5, SD = 1.98, and *p* < 0.001) (Figure 4A) and the subjectively reported difficulty ratings were significantly lower on the low (mean = 3.26, SD = 1.09) compared to the high cognitive load condition (mean = 7.09, SD = 1.03, and *p* < 0.001) (Figure 4B).

The event related analysis of our SCR data showed a significant main effect [*F*_(1,30)_ = 13.834, *p* = 0.001] of the cognitive load on the Global Mean values in the 10 s after odor application. The Global Mean values in this response window during the low cognitive load setting (mean = 4.07, SD = 4.89) were lower than in the high cognitive load setting (mean = 4.20, SD = 4.98, and *p* = 0.001) (Figure 4C).

## Discussion

In this study, we aimed to test the influence of cognitive load on odor intensity perception of food and non-food odors in healthy adults. Our results indicate that an everyday task like playing a video game creates a cognitive load that is sufficient to influence the way we perceive odors. The intensity rating for food odors as well as for non-food odors decreased with higher cognitive load. An explanation for this effect could be that higher cognitive load lowers the attentional capacity for smelling odors. This is consistent with the findings of Hoffmann-Hensel et al. (2017) and extents the results with regards to a more ecologically valid stimulation via a Tetris game and with regards to different odors. Hoffmann-Hensel et al. (2017) observed that a high cognitive load task lowers the activity in olfactory brain structures which also explains the decrease in odor intensity ratings under a high cognitive load condition. These recent results are also consistent with the findings of Lau-Zhu et al. (2017) who discovered that playing Tetris competes with other tasks for working memory which can lead to a different way information is processed.

The studies by Bolhuis et al. (2011); Ramaekers et al. (2014), and Jayasinghe et al. (2017) showed that a high taste intensity results in a lower food intake. Additionally, van der Wal and van Dillen (2013) indicated in their study that a lower gustatory intensity perception leads the subjects to choose taste solutions with higher concentrations and to consume more of these taste solutions. Based on our results and because of the important role olfaction has on taste and food consumption (Murphy et al., 1977), we expect that the decreased gustatory intensity perception in the experiment by van der Wal and van Dillen (2013) is related to a decrease in olfactory intensity perception during cognitive load tasks which we were able to show in our experiment. Based on our findings, we believe that the lower olfactory intensity perception may be one of the reasons why people consume more food while watching TV as observed by Braude and Stevenson (2014).

We further established that subjects with a higher BMI tend to rate the intensity of odors lower than those with a lower BMI. This is coherent with the findings of Peng et al. (2019) who showed that the body weight is negatively correlated with olfactory ability. Our group of subjects contained mostly people with a normal BMI but also people with a BMI over 25 which indicates overweight (Weir and Jan, 2019). Future studies should use subjects with obesity to investigate the cause of decreased intensity perception in subjects with a high BMI.

The number of rows solved, and the self-reported difficulty ratings are consistent with our defined cognitive load conditions. The participants were able to solve significantly less rows in the same time (Figure 4A) and rated the difficulty of the game significantly higher (Figure 4B). Moreover, the SCR data analysis indicates that the high cognitive load condition reliably delivered a high cognitive load based on the effect described by Shimomura et al. (2008) (Figure 4C).

Previous studies investigating the influence of cognitive load on taste or olfactory perception used cognitive tasks like memorizing a combination of consonants (van der Wal and van Dillen, 2013) and investigated gustation without using complex taste percepts (van der Wal and van Dillen, 2013). However, food is usually a complex mixture of different aromas and tastes so these results may not transfer to real-life situations. Future studies should also use everyday activities like playing videogames or watching videos as a cognitive task like we did in our study and complex sensory stimuli to render the setting more comparable to real-life situations.

### Limitations

Since, we selected two odors per odor category (“banana” and “orange” for food, “leather” and “wood” for non-food) it is hard to draw conclusions for each of these categories in general. More food odors should be considered in the future. The same counts for non-food odors. The food odors one should consider for future studies could also represent foods that are generally classified as “healthy” and “unhealthy” or odors that represent “snacks” and “full meals.” The non-food odors could represent “natural” objects and “artificial” objects.

Our study did not let the participant choose a specific odor while receiving a specific cognitive load but tried to clarify the basic influence of a cognitive load on odor perception. Future studies should investigate if the decreased intensity perception leads to a different behavior in choosing odors. Such a study could reveal what influence everyday activities like watching TV or playing video games may have on our food choices.

While our study showed that playing video games like Tetris is sufficient to create a cognitive load that leads to a decreased odor intensity perception, we did not investigate if playing Tetris also increases the food intake. Future studies should try to include the measurement of snack size while playing video games.

We only investigated the influence of cognitive load on odor intensity but not on odor pleasantness or odor familiarity. In a future study one should explore if a high cognitive load also alters these odor characteristics and how this effect alters food choices and food intake.

In our study, we only asked our subjects for the intensity ratings but did not measure a physiological value for the odor intensity perceptions. Future studies could use magnetic resonance imaging (MRI) and functional MRI (fMRI) like Hoffmann-Hensel et al. (2017) did in their study to investigate the neuronal activity responsible for the processing and perception of scents and the influences of cognitive load on this network.

We did not monitor the breathing pattern of our subjects during the experiment. Doing so could have assured a more targeted application of our odors. Furthermore, we would have been able to detect changes in the breathing patterns between low and high cognitive load, which may have an impact on the perceived odor intensities. Future studies should monitor the subjects breathing patterns to synchronize the odor application and to look for effects on odor intensity.

In our pilot study a different method for odor application was used. Future studies should try to use the same method for establishing similarly intense odor concentrations and for the actual study to make them more comparable.

## Conclusion

In conclusion, a higher cognitive load leads to a decreased intensity perception for edible odors as well as for inedible odors. This may explain the increased food intake while watching TV or playing video games. Future studies should use multisensory paradigms to investigate if the decreased intensity perception leads to different food choices in situations with a high cognitive load. Those studies should also use a modern everyday scenario like watching TV or playing video games.

Based on our results we recommend to not engage with digital media such as watching TV or playing video games while eating for a healthier way of food consumption.

## Data Availability Statement

The raw data supporting the conclusions of this article will be made available by the authors upon request via email.

## Ethics Statement

The studies involving human participants were reviewed and approved by the RWTH University Aachen Medical Faculty local ethics board. The patients/participants provided their written informed consent to participate in this study.

## Author Contributions

SS acquired and analyzed the data, interpreted the results, wrote the manuscript, had full access to all data in the study, and took the responsibility for the integrity and accuracy of data analysis. LH programmed the Tetris task in Psychopy, helped with the data analysis, and revised the manuscript. RR-R interpreted the results and revised the manuscript. JF was the guarantor of this work, supervised the study, interpreted the results, and revised the manuscript. All authors approved the final article.

## Conflict of Interest

JF is part of Fraunhofer IVV as stated in the affiliations section. The remaining authors declare that the research was conducted in the absence of any commercial or financial relationships that could be construed as a potential conflict of interest.

## Footnotes

1 https://johnloeber.com/docs/tetris.html

2 http://www.ledalab.de
